# Smoking Bans and Circulatory System Disease Mortality Reduction in Macao (China): Using GRA Models

**DOI:** 10.3390/ijerph20054516

**Published:** 2023-03-03

**Authors:** Xinxin Peng, Xiaolei Tang, Jing Hua Zhang, Yijun Chen

**Affiliations:** 1 School of Business, Macao University of Science and Technology, Macao 999078, China; 2 School of Management, Jiangsu University of Technology, Changzhou 213001, China; 3 Faculty of Social Sciences, University of Macau, Macao 999078, China

**Keywords:** smoking ban, circulatory system disease mortality, grey relational analysis, Macao

## Abstract

This study evaluates the association between smoking rates and mortality from circulatory system diseases (CSD) after implementing a series of smoking bans in Macao (China). (1) Background: Macao phased in strict total smoking bans since 2012. During the past decade, smoking rates among Macao women have dropped by half. CSD mortalities in Macao also show a declining trend. (2) Method: Grey relational analysis (GRA) models were adopted to rank the importance of some key factors, such as income per capita, physician density, and smoking rates. Additionally, regressions were performed with the bootstrapping method. (3) Results: Overall, smoking rate was ranked as the most important factor affecting CSD mortality among the Macao population. It consistently remains the primary factor among Macao’s female population. Each year, on average 5 CSD-caused deaths were avoided among every 100,000 women, equivalent to about 11.45% of the mean annual CSD mortality. (4) Conclusions: After the implementation of smoking bans in Macao, the decrease in smoking rate among women plays a primary role in the reduction in CSD mortality. To avoid excess CSD mortality due to smoking, Macao needs to continue to promote smoking cessation among the male population.

## 1. Introduction

Tobacco smoking has been well established as an independent, modifiable risk factor for premature mortality of several medical causes such as coronary, cerebral, and peripheral arterial diseases [1,2]. Cardiovascular diseases have been the leading cause of death worldwide associated with smoking [3,4]. While heavy smoking is equally hazardous to both genders, women smokers have been found to be at greater risk of smoking-related cardiovascular diseases, such as coronary heart disease [3,5], ischemic heart disease [6], and acute myocardial infarction (AMI) [7,8,9].

The WHO (World Health Organization) Framework Convention on Tobacco Control (WHO FCTC) has been recognized as the most powerful tool to counter tobacco’s negative impacts. Smoking bans are supported by WHO on the grounds that they improve health outcomes by lowering exposure to second-hand smoking (SHS) [10] or third-hand smoke (THS) [11] and potentially reducing the number of smokers. Smoking cessation by the individual is an effective measure to reduce cardiovascular diseases, also helping to reduce the economic burden of healthcare [12,13,14,15,16,17,18]. Overall, women smoke less than men, but it is noteworthy that despite significant tobacco control efforts, women’s smoking rates have barely changed and in some countries have even increased [19]. Around the world, increasingly young women (under 25 years old) are smoking tobacco [19]. Targeting tobacco smoking was especially predicted to reduce premature cardiovascular disease mortality among women in high-income Asia-Pacific and Western Europe regions [20].

### 1.1. Tobacco Smoking in China

China is the country with the greatest tobacco consumption in the world [18]. In 2018, the smoking rate among the population over the age of 15 in China was 26.6%, of which the male smoking rate was 50.5%. The size of the smoker population in China exceeds 300 million. It is estimated that more than 1 million people in China lose their lives due to tobacco smoking every year and the mortality may increase to 2 million per year by 2030 if no effective action is taken [21].

Some major central cities and provincial capital cities in China have taken strict actions in recent years to adopt smoke-free laws or implement comprehensive tobacco control actions [15,17,22]. Smoking is prohibited in all indoor workplaces, indoor public places, and public transportation in 9 of the 21 Chinese cities that have enacted smoke-free laws [22].

### 1.2. Health System and Smoking Bans in Macao (China)

The Macao Special Administrative Region of China (“Macao”) is located on the Pearl River Delta on the southeast coast of mainland China. With a total population of about 671,900 [23], Macao has the world’s highest population density of 20,620 persons per square kilometer [24]. With a life expectancy of 84.98 years [25], people over the age of 65 account for 12.1% of the population [24].

Macao’s health system is a hybrid one, in which a public health provider has a key role and some private ones play supplementary roles [26]. Circulatory system diseases are the second leading cause of death in Macao, accounting for about 23.9% of total deaths in 2021 [27].

Macao has grown into a major international resort city and a top destination for gambling tourism, accounting for 60% of the local GDP and 70% of local tax revenue. In 2019, the total number of workers in the six tourism satellite industries (gaming, retail trade, food and beverage, hotel, passenger transportation, and travel agency services) was approximately 203,000, accounting for nearly half of Macao’s working population [28]. In particular, more than twenty percent of Macao’s working population is in the gaming industry, which is infamous for heavy smoking and poor indoor air quality. It was estimated that, when there were no smoking bans, each year, 20 percent of local deaths were caused by smoking [29].

Despite strong resistance from the gaming industry [30], during the past decade, the Macao government has put significant efforts into establishing a smoke-free local environment through a variety of approaches, including legislation, law enforcement, health education, and smoking cessation aid. Smoking bans were phased in from 2012 to ensure that indoor air quality meets safety standards, protecting the health of residents and visitors alike. A partial ban with casinos as the exception in January of 2012, a full smoking ban allowing smoking lounges in local casinos in October 2014 [30], and later a blanket smoking ban without smoking lounges after 2018 were implemented in Macao [31]. Virtually all forms of tobacco advertising and promotion through any medium are prohibited.

The Macao government has demonstrated firm determination and action to enforce these smoking bans. From 2018 to 2020, tobacco control law enforcement officers of Macao inspected a total of 859,000 locations, and the total case number of prosecutions for smoking ban offenses reached 13,300 [32]. The maximum fine for smoking offenses was raised from USD 75 equivalent to USD 188. The Health Bureau of Macao provides free clinical services for smoking cessation. In August 2022, an amendment to the smoking ban was passed in Macao, prohibiting the manufacturing, transporting, distribution, importing, or exporting of e-cigarettes in and out of Macao. The retail sale, advertising, or promotion of e-cigarettes is prohibited.

**Figure 1:**
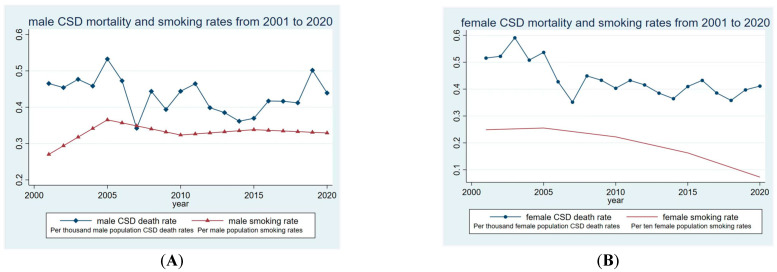
Time trend patterns of circulatory system disease mortality and smoking rates in Macao S.A.R., China (2001–2020). Panel (**A**): male; panel (**B**): female.

Figure 1 below displays the time trend patterns of CSD mortality and smoking rate among male and female residents in Macao over the past 20 years, respectively. The overall smoking rate among the Macao population aged 15 years and above decreased from 33.7% in 2011 (upon the implementation of the smoking ban) to 11.2% in 2020 [33].

As displayed in Panel A of Figure 1, neither the curve of male smoking rates nor that of the male CSD mortality rates have a clear tendency. In Panel B of Figure 1, the curve of female CSD mortality rates demonstrates an observable declining tendency and is associated with an apparently declining trend in smoking rates.

The literature providing empirical evidence of the beneficial impacts of a smoking ban on circulatory system diseases is rich, especially cardiovascular diseases [4,14,15,16,17,34,35,36]. However, only a small amount of the literature examines empirical health evidence regarding smoke-free policies in China [15,17]. Currently, there has been no empirical study examining the health outcome of smoking bans in Macao during the past decade. Meanwhile, due to confounding factors such as health technology advancements in the treatment of underlying diseases, promotion of healthy lifestyles, and preventive medicine, smoking bans’ effects on health outcomes in major cities or nationwide may not be significant in empirical studies [37].

### 1.3. Aims of this Study

Applying grey relational analysis (GRA) models, this study aimed to assess the contribution of smoking bans to the decline of circulatory system diseases in Macao (China). GRA assumes a non-functional sequence model and does not generate results in conflict with qualitative analysis; its advantages include being computationally simple and not requiring large amounts of data or data normalization [38,39]. This method can be flexibly applied to various fields [38,40,41].

The findings of this study may provide an empirical evaluation of smoking bans in Macao and policy suggestions for the next stage. The empirical evidence on the positive effects of smoking bans on health outcomes will serve as a policy reference for promoting a comprehensive smoke-free policy, even among the tourism and hospitality sectors in China and other Asian nations.

## 2. Research Methods

A series of grey relational analysis (GRA) models were applied in this study to examine the role of smoking rate in reducing CSD mortalities. Ordinary least-squared (OLS) regression analysis was performed to estimate the size of the association. Due to the small observation numbers in this sample (*n* = 40), a bootstrapping method (with a repetition of 1000 times) was adopted to generate robust standard errors.

### 2.1. Grey Relational Analysis (GRA)

Also known as Deng’s grey incidence analysis, GRA models are based on grey system theory [40,42,43,44,45]. Grey system theory is based on the recognition and realization that all-natural and social systems are intrinsically uncertain and subject to a variety of uncertainties and noises, which are caused by internal or external disturbances as well as the limitations of human knowledge and perception. Discrepancies in the system or the available data is one of the defining qualities of an uncertain system [45]. Generally, imperfection or inaccuracy of information can be categorized into three types based on its origin (namely conceptual, level of perspective, and prediction inaccuracies). For instance, phrases, such as “large”, “small”, “fat”, “thin”, “good”, “bad”, “young”, and “beautiful”, are commonly used, but they are subjective and lack a precise meaning [45]. A system with imperfect information is represented as being midway between a white system (with perfect information) and a black system (with zero information) [45].

Based on the intuition introduced above, GRA analyzes the degrees of geometric curve similarity between data sequences. If the curve similarity is higher, the data sequences are judged to be of higher relevance, and vice versa [42,45]. In this way, a GRA model reflects the interactions between the factors examined based on the correlation coefficient of points. Specifically, Deng’s GRA mode follows the computational steps below [40,42,43,44,45,46,47].

Step 1 is to construct the reference sequence *x*_0_ (the dependent variable) and the comparison sequence *x_i_* (*i* = 1, 2, 3…, *n*), which serves as the independent variables [48].

Step 2 is to calculate γ*_i_* (*k*), given observation number *k* (*k* = 1, 2… *m*), according to Equations (1)–(3). γ*_i_* (*k*) is also called a grey relational coefficient.

(1) $$\gamma_{i}\left(x_{0}\left(k\right),x_{i}\left(k\right)\right)=\frac{\min\min|{x_{0}}^{\prime }\left(k\right)-{x_{i}}^{\prime }\left(k\right)|+\varepsilon \max\max|{x_{0}}^{\prime }\left(k\right)-{x_{i}}^{\prime }\left(k\right)|}{\left|{x_{0}}^{\prime }\left(k\right)-{x_{i}}^{\prime }\left(k\right)|+\varepsilon \max\max|{x_{0}}^{\prime }\left(k\right)-{x_{i}}^{\prime }\left(k\right)\right|}\;$$

where

(2) $$x_{i}^{\prime }\left(k\right)=\frac{x_{i}\left(k\right)}{\bar{x_{i}}\left(k\right)},$$

(3) $$\bar{x_{i}}\left(k\right)=\frac{1}{n}\overset{n}{\underset{k=1}{\sum }}x_{i}\left(k\right)$$

*i* = 1, 2…, *n*;

*k* = 1, 2…, *m*; (*k* indicates observation time or observation number).

In Equation (1), *ε* has a value between 0 and 1, and the middle value is often assumed to be 0.5 [42,49]. *ε* is also called the resolution coefficient.

Step 3 uses the results of *γ_i_* (*k*) from Step 2 to calculate *β_i_* (*k*), the grey relational degree, as in Equation (4). *β_i_* (*k*) is also called Deng’s degree of grey incidence.

(4) $$\beta_{i}\left(k\right)=\frac{1}{n}\overset{N}{\underset{k=1}{\sum }}\gamma_{i}\left(k\right)$$

The correlation degree is interpreted as a ranking order. The parameters for the GRA models range from 0–1, with it being regarded as strongly associated if it is close to 1 and frailly associated if it deviates from 1 [42,49,50]. The higher the correlation degree is, the higher the ranking is [47]. In this way, the grey relational degree reflects the inter-influences among the factors analyzed [43,45,48]. To justify the comparison of coefficients in GRA, each variable of the original data is standardized, removing the units of measurement before calculating the correlation degree.

Javed et al. [42,49,51] and Liu et al. [45] further developed and refined GRA models with computing details, including several types of “grey relational degree” numbers (or “degree of grey occurrence”). While absolute GRA utilizes the specific point grey and analyzes the correlations between factors, Relative GRA utilizes the integral visual angle [45]. Calculating the average value of absolute GRA and relative GRA, the SDGRA reflects the line of similar degree compared to the proximity of the pilot’s rate of change. It is a comprehensive indicator of sequence relationships [45]. Further, the SSGRA model was developed by calculating the average value of Deng’s GRA and absolute GRA. SSGRA has the advantage of reflecting “overall closeness between two sequences based on particular points and integral perspectives” [49].

In summary, Deng’s GRA model is typically regarded as the baseline model, while SDGRA and SSGRA models’ estimations are preferred to absolute GRA and relative GRA models [49].

### 2.2. Advantages of GRA Models

When compared to traditional statistical inference models, GRA models have several advantages for decision making [51,52]. First, GRA can effectively provide meaningful inference even with missing, insufficient, or incomplete data or with uncertainties and incomplete information [51,53].

Second, unlike statistical and probability theory models, GRA models do not need a normal distribution assumption or a big sample size [45,54]. With only a small amount of data, GRA can reliably identify key factors based on the relationship between the reference series and the comparability series data [50]. The dataset available in this study consists solely of aggregated annual disease mortality rates and smoking rates from the past 20 years, which are insufficient for performing traditional regression analysis directly. Despite their data limitations, GRA models can yield meaningful analysis results.

Third, in many decision or policy making scenarios, the order of relationship closeness determined by the grey relation degree is frequently more appropriate to use than the precise numerical values of the estimated coefficients. [51,53]. In this study, it was meaningful to obtain ranking information about the importance of smoking rates among multiple confounding factors, which might also contribute to the reduction in CSD mortality.

In addition to engineering [55], management [52], and environmental science [56], GRA models have been adopted in healthcare management studies to evaluate patient satisfaction [42,49], healthcare service quality [57,58], performance [59], efficiency [60,61], healthcare resource allocations [62,63], etc.

### 2.3. Ordinary Least Squared (OLS) Regression Analysis with Bootstrapping

OLS regression analysis was performed based on the relevant variables identified using GRA models. The Ramsey regression equation specification error test (RESET) was performed as a diagnostic test of regression specification error for potentially omitted variables.

To address the issue of a small sample size, we adopted the bootstrap method to generate a robust standard error. The bootstrapping method is valid for a small sample because it is a nonparametric approach for evaluating the distribution of statistics-based on random resampling [64]. Unlike a traditional parametric approach, the bootstrapping method does not depend upon strong distributional assumptions of a sample (such as i.i.d. or normal distribution). Instead, it estimates the asymptotic covariance matrix by random sampling from the empirical distribution [65].

### 2.4. Data Analysis

The baseline model of GRA in this study was Deng’s GRA model, in which the arithmetic mean is taken as the initial point [54]. SDGRA and SSGRA models were the main models. Absolute and Relative GRA were performed only for reference.

The three explanatory variables included income per capita, physician density, and smoking rate, including observations from 2000 to 2020. Explanatory variables with observations lagged one year were examined as a robustness check. Extra robustness checks included rate of alcohol use and rate of overweight from 2000 to 2015 as explanatory variables.

The Gray Level Correlation Software 7.0.1 (Grey System Research Institute, Nanjing, China) (available at http://igss.nuaa.edu.cn, accessed on 18 December 2022) was adopted to perform Deng’s, absolute, relative, and SDGRA models. The SSGRA model was computed using Microsoft Excel (Version 2002).

The Stata 14 statistical package (Stata Corp LP, College Station, TX, USA) was used to perform OLS regression analysis.

## 3. Data and Variables

### 3.1. Data Sources and Ethical Declaration of the Data

Annual data on income per capita and physician density from the year 2001 to 2020 were obtained from the Statistics and Census Service of Macao. Annual data on resident smoking rates were collected from Macao Health Bureau and Macao Sports Bureau.

Residents’ alcohol consumption rates and obesity data were obtained from the Macao Citizen Physical Fitness Monitoring Report (2001, 2005, 2010, and 2015), which was sponsored and published by the Sports Bureau of the Macau Government. The original data of alcohol consumption rates and obesity rates comprised discrete points within the years 2001, 2005, 2010, and 2015 from four waves of the survey.

Using only the publicly available statistics data disclosed by government departments, this study did not collect any person’s data. There were no experimental designs used in this study, nor were any patients or survey respondents involved. Therefore, this study did not require extra ethics approval.

### 3.2. Dependent Variables

The mortality rate of circulatory system disease (CSD) (ICD10: I00–I99) [66] from 2001 to 2020 was analyzed in this study. CSD in the ICD10 includes: acute rheumatic fever (I00–I02); chronic rheumatic heart diseases (I05–I09); hypertensive diseases (I10–I15); ischemic heart diseases (I20–I25); pulmonary heart disease and diseases of pulmonary circulation (I26–I28); other forms of heart disease (I30–I52); cerebrovascular diseases (I60–I69); diseases of arteries, arterioles, and capillaries (I70–I79); diseases of veins, lymphatic vessels and lymph nodes, not elsewhere classified (I80–I89); and other and unspecified disorders of the circulatory system (I95–I99).

In Macao’s population, cardiovascular and cerebrovascular diseases account for more than 90% of the deaths in the category of CSD [27]. Rates of the total population, male citizens, and female citizens over the studied period were analyzed separately.

### 3.3. Independent Variables

(1) Income per capita. Income can influence cardiovascular events or death through several interconnected routes. For instance, high-income communities are more likely to understand hypertension prevention and control and have better access and adherence to medical care. Healthy lifestyle choices also differ according to income levels [67].
(2) Physician density was measured as the number of physicians per population of 1000. It is an important indicator of the primary care supply [68].
(3) Smoking rate was calculated as the ratio of current adult smokers (aged 18 years or older) to the population. A current smoker was defined as someone who reported smoking every day while participating in the Macao Citizen Physical Fitness Monitoring Report survey.
(4) Alcohol usage rate was calculated as the percentage of the adult population who reported drinking at least once every week when responding to the survey for the Macao Citizen Physical Fitness Monitoring Report. Epidemiological studies have found a J-shaped relationship between alcohol use and cardiovascular problems [69]. Public health experts have warned that the only safe alcohol consumption level is zero consumption [70,71].
(5) Rate of overweight and obesity indicated those either overweight or obese among those aged 40 and older. According to the WHO standard, a BMI (body mass index) in the range of 25 to 30 is defined to be overweight, while a BMI greater than 30 is defined as obese. Overweight and obesity in China have significantly increased over the past few decades. For adults in 2015–2019 China, 34.3% were overweight and 16.4% were obese [72]. Obesity is associated with increased cardiovascular mortality in China [73,74].

## 4. Results

### 4.1. Descriptive Statistics of the Data

Table 1 below reports the descriptive characteristics of key variables analyzed in this study. As reported in Panel A, during the studied period, CSD mortality in Macao was 86.9 per 100,000 people, with similar levels among both male and female populations. The density of physicians was about 2.4 per 1000 people in Macao.

While the population smoking rate was about 15% on average during the study’s period, the smoking rate was as high as 33% among men, in contrast to about 2% among women in Macao.

As reported in Panel B of Table 1, the rate of alcohol use among male residents in Macao during the studied period is 46.2%, which is about 3.4 times the female rate. While the male rate of alcohol use in Macao had only a moderate increase of about 2.68 percentage points, the female rate of alcohol use in Macao increased from 10.2% in 2001 to 16.96% in 2015. In contrast to the stable overweight and obesity rate among women aged 40 and over, the rate among men increased by about 15.6 percentage points from 29.1% in 2001 to 44.7% in 2015.

### 4.2. Results of GRA Models

GRA models were applied to analyze the data, ranking the importance of the relevant factors of CSD mortality in Macao from 2001 to 2020. As reported in Table 2, the baseline model results show that, for the male population in Macao, physician density and smoking rates are ranked as the most important determinants. The results are consistent among all GRA models. For the female population, physician density and smoking rates are also important determinants, while Deng’s GRA and SDGRA models show that the women’s smoking rate is ranked as the most important one. The ranking pattern of the total population largely is a mixture.

Considering the time-lag effects of physician density (primary care) and smoking rates, we further lagged the explanatory variables for a one-year period and performed the same analysis. As reported in Table 3, the GRA ranks of relevant male CSD mortality factors are consistent with the baseline mode. As for the female population, all models, except the relative GRA model, consistently estimated smoking rate as no. 1. In particular, Deng’s GRA, SDGRA, and SSGRA models were found to be the most comprehensive and effective evaluation models.

For robustness checking, GRA tests were expanded by adding two extra variables: the rate of alcohol use and the rate of overweight and obesity among people aged 40 and older from 2001 to 2015. As reported in Table 4, the GRA models suggest that, for the male population, rate of alcohol use and physician densities are the leading factors relevant to CSD mortalities during the study period. For female CSD mortalities, smoking rate is consistently rated as the most significant relevant factor, followed by the risk factor of overweight and obesity (Aged >40).

### 4.3. Regression Analysis of CSD Mortality in Macao

Based on the GRA results, we also performed regression analysis on CSD mortalities and relevant factors as shown in Table 5. The dependent variable was the annual CSD mortalities, which included 40 observations from the male and female population. Smoking rate and physician density were included as two explanatory variables because these two factors are rated by GRA methods as the most important factors. A dummy variable of “female after smoking ban” was generated to indicate the female observations after the implementation of the smoking ban in 2012 (included). The estimated coefficient of this variable captured the excess changes in female mortality after the implementation of the smoking ban.

Column (1) reports the regression analysis results of the baseline model. While physician density has a highly significant negative association with CSD mortality, the coefficient of smoking rate is insignificant. This may be mainly due to the insignificant effects of smoking rates among men.

Column (2) is the full model, including the variable of interest “female after smoking ban”. As reported in Column (2), while an increase in physician density (1 per 1000 people) is significantly associated with a reduction of about 8.95 CSD deaths per 100,000 people, the CSD mortality in Macao after the implementation of the smoking ban in 2012 has an extra annual reduction of 5 deaths for every 100,000 women. In addition, the R-squared is 0.283, which is the highest one among the three specifications tested.

Column (3) reports results without including the smoking rate in the regression, and the results are robust.

The bottom line of Table 5 reports the results of the Ramsey RESET test, which indicates no evidence of omitted variables in all three specifications.

## 5. Discussion

Applying GRA models, this study examined the key factors associated with CSD mortalities in Macao (China) from 2001 to 2020. The findings of this study based on GRA models indicate that smoking rate is consistently the most important factor associated with women’s CSD mortality in Macao, while physician density was ranked as the second most important factor. In contrast, for men’s CSD mortality in Macao, the physician density was estimated as the most important factor, while smoking rate has a secondary role.

These findings suggest that women in Macao may have obtained substantial health benefits from smoking bans, while men had few. Two major reasons for this difference may be considered. First, women may have directly benefited from their own smoking cessation encouraged and supported by public health policies, as evidenced by a significantly lower smoking rate following the smoking bans [18]. Second, after full smoking bans in the local community, women usually have extra health benefits from less exposure to secondhand smoke (SHS) [16]. In particular, decreased exposure to SHS among nonsmokers may result in a decrease in myocardial infarctions [14].

This study’s estimate of the smoking ban’s health outcome effect size is comparable to those that have been reported for mainland China. This study estimates that, owing to the smoking bans in Macao since 2012, on average each year about 5 CSD deaths were avoided among every 100,000 women, equivalent to about 11.45% of the mean annual CSD mortality (43.66 per 100,000 people), or about 16.8 CSD deaths among the Macao female population. A study of Beijing’s 2015 tobacco control policy package estimated that the associated drop in hospital admissions for cardiovascular diseases was overall more than 10% [75]. In another study, Zheng et al. estimated reductions in hospital admissions were about 5.4% of AMI and 5.6% of stroke cases [76]. Additionally, the increasing trend in stroke admission events was reduced by 15.3% [76]. Research studying Tianjin (China) found the mortality rate from AMI decreased by 16% per year, while the mortality rate of stroke among those under 35 decreased by 2% annually after the implementation of smoke-free legislation [17].

Meanwhile, the findings of this study indicate that Macao’s smoking bans did not successfully achieve their health goals among the male population. The full smoking ban in Macao’s casinos was expected to help many casino workers quit smoking. According to a casino employee survey in 2008 (before the implementation of the initial smoking ban in Macao), more than half of the respondents (*n* = 315, men = 165, 52.4%) reported that they would try to quit smoking if smoking was outlawed at work [77]. Nevertheless, despite the eventual implementation of the smoking ban, the men’s smoking rate in Macao did not significantly decline and the associated mortality could not be avoided.

In addition, the findings of this study also reveal that alcohol using may be among the leading risk factors for CSD mortality among men in Macao. This is a similar health concern among men in mainland China [78], and most Chinese people are unaware of the rigorous evidence-based public health warning that zero consumption is the only safe alcohol usage level.

This study has several limitations. First, the analysis of this study is largely limited by the availability of the data. An overall reduction in female mortality and smoking rates was observed, but no information is available regarding associated societal health inequalities, such as the differences between lower and higher SES groups. Second, the GRA method has a limitation in that GRA models with different estimation approaches may sometimes produce different analysis results. The ranking orders predicted by GRA models lack precise numerical values, such as the number of deaths avoided, for further policy impact analysis. To address concerns about the gender gap in smoking cessation, future research should focus on identifying male-specific smoking cessation barriers in the context of complete smoking bans. Whereas internal barriers, such as stress and cravings, emerged to be more prominent in women, external barriers, such as the widespread availability of cigarettes and the social aspects of smoking, were more prevalent in men [79].

## 6. Conclusions

Applying GRA models, this study found that smoking rate is rated as the most important factor associated with women’s CSD mortality in Macao between the years of 2001 and 2020. Women’s CSD mortality decreased annually by 11.45% as a result of smoking cessation, but there were no comparable results for men.

The findings of this study have significant public health policy implications for Macao. Although smoking bans have been successfully implemented in the city’s casinos, Macao nevertheless needs to focus on reducing the male smoking rate and promoting a healthy lifestyle. It is recommended that mainland China and other emerging economies with high smoking rates adopt total smoking bans and strong legal enforcement. Additional steps, including health education and cessation support services, should be taken to further realize smoking rate reduction in high-risk population groups.

## Figures and Tables

**Table 1:** Descriptive characteristics of mortalities of circulatory system diseases and relevant factors 2001–2020, Macao.

**Panel A: Descriptive Characteristics of Mortalities of Circulatory System Diseases and Relevant Factors (Years 2001–2020, Macao)**					
	**Mean**	**Std. Err.**	**[95% Conf.**	**Interval]**	
Total CSD mortality (per 100k people)	86.91	2.33	82.04	91.77	
Male	43.25	1.09	40.97	45.52	
Female	43.66	1.46	40.60	46.71	
Income per capita (1k MOP)	371.64	38.82	290.39	452.88	
Physicians (per 1k people)	2.44	0.04	2.35	2.53	
Smoking rate (population) (%)	15.12	0.15	14.80	15.44	
Male	33.08	0.45	32.13	34.03	
Female	1.96	0.13	1.68	2.24	
**Panel B: Descriptive characteristics of factors (The years 2001 to 2015, Macao) ^1^**					
	**2001**	**2005**	**2010**	**2015**	**Average**
Rate of alcohol use (population) (%)	27.72	27.17	27.01	29.55	27.86
Male (%)	44.02	46.92	47.32	46.68	46.23
Female (%)	10.21	13.25	13.87	16.96	13.57
Overweight & obesity rate (>=40 years) (%)	33.70	40.20	40.20	43.00	39.30
Male (%)	29.10	43.60	46.20	44.70	40.90
Female (%)	38.60	37.80	36.40	41.80	38.70

Note: ^1^ Only data from 2001 to 2015 were available. These data were only included for robustness check.

**Table 2:** Grey relation degree and ranking of circulatory system disease mortality relevant factors (the years 2001–2020, Macao).

	Deng’s GRA	Rank	SDGRA	Rank	SSGRA	Rank	Absolute	Rank	Relative	Rank
Male										
Income per capita	0.567	3	0.512	3	0.534	3	0.500	3	0.524	3
Physician density	0.885	1	0.645	1	0.727	1	0.570	1	0.720	1
Smoking rate	0.874	2	0.599	2	0.690	2	0.505	2	0.693	2
Female										
Income per capita	0.566	3	0.522	3	0.533	3	0.500	3	0.544	3
Physician density	0.859	2	0.765	1	0.742	2	0.624	1	0.906	1
Smoking rate	0.941	1	0.746	2	0.770	1	0.599	2	0.893	2
Total										
Income per capita	0.565	3	0.517	3	0.533	3	0.500	3	0.534	3
Physician density	0.872	2	0.740	1	0.767	2	0.663	2	0.818	1
Smoking rate	0.948	1	0.700	2	0.8713	1	0.794	1	0.605	2

**Table 3:** Grey relation degree and ranking of circulatory system disease mortality relevant factors (the years 2001–2020, Macao) (with time lag effects).

Lagged Variable ^1^	Deng’s	Rank	SDGRA	Rank	SSGRA	Rank	Absolute	Rank	Relative	Rank
Male										
Income per capita	0.567	3	0.512	3	0.534	3	0.500	3	0.524	3
Physician	0.892	1	0.656	1	0.734	1	0.575	1	0.737	1
Smoking rate	0.880	2	0.604	2	0.692	2	0.505	2	0.702	2
Female										
Income per capita	0.566	3	0.522	3	0.533	3	0.500	3	0.544	3
Physician	0.860	2	0.785	2	0.747	2	0.634	1	0.936	2
Smoking rate	0.942	1	0.791	1	0.780	1	0.619	2	0.963	1
Total										
Income per capita	0.565	3	0.517	3	0.533	3	0.500	3	0.534	3
Physician	0.874	2	0.759	1	0.775	2	0.676	1	0.842	1
Smoking rate	0.945	1	0.626	2	0.801	1	0.656	2	0.596	2

Note: ^1^ mortality rate at year *t* was estimated using explanatory variables at year *t − 1*.

**Table 4:** Grey relation degree and ranking of circulatory system disease mortality relevant factors (years 2001–2015, Macao).

	Deng’s	Rank	SDGRA	Rank	SSGRA	Rank	Absolute	Rank	Relative	Rank
Male										
Income per capita	0.626	5	0.516	5	0.563	5	0.500	5	0.533	5
Physician	0.897	2	0.681	2	0.745	1	0.593	1	0.769	2
Smoking rate	0.875	3	0.605	3	0.690	3	0.506	3	0.704	3
Rate of alcohol use	0.936	1	0.726	1	0.725	2	0.513	2	0.939	1
Overweight and obesity rate (aged >40)	0.800	4	0.555	4	0.651	4	0.503	4	0.608	4
Female										
Income per capita	0.624	5	0.525	5	0.562	5	0.500	5	0.550	5
Physician	0.878	3	0.776	2	0.758	3	0.638	2	0.913	1
Smoking rate	0.967	1	0.790	1	0.831	1	0.695	1	0.884	2
Rate of alcohol use	0.824	4	0.617	4	0.669	4	0.515	4	0.719	3
Overweight and obesity rate (aged >40)	0.933	2	0.624	3	0.768	2	0.603	3	0.644	4
Total										
Income per capita	0.625	5	0.521	5	0.563	5	0.500	5	0.542	5
Physician	0.889	3	0.764	2	0.786	3	0.683	3	0.845	1
Smoking rate	0.949	1	0.744	3	0.897	2	0.845	2	0.643	3(4)
Rate of alcohol use	0.942	2	0.812	1	0.961	1	0.980	1	0.643	3(4)
Overweight and obesity rate (aged >40)	0.883	4	0.665	4	0.697	4	0.511	4	0.818	2

**Table 5:** Regression analysis of CSD mortality in Macao (years 2001–2020). (Dep. var. = CSD mortality per 100 k people).

Variables	(1)	(2)	(3)
Smoking rate (%)	−0.83	−8.346	
	(5.14) ^1^	(8.11)	
Physician density	−13.88 *** ^2^	−8.95 *	−11.11 **
	(4.32)	(4.66)	(4.82)
Female after ban		−5.024 **	−2.872 *
		(2.53)	(1.54)
Constant	77.46 ***	67.88 ***	71.19 ***
	(11.13)	(10.99)	(11.86)
Observations	40	40	40
R-squared	0.21	0.28	0.25
Ramsey RESET test	F(3, 34) = 2.12	F(3, 33) = 1.17	F(3, 34) = 1.88
	Prob > F = 0.1157	Prob > F = 0.3359	Prob > F = 0.1509

Note: ^1^ Bootstrapped (with repetition of 1000 times) standard errors in parentheses. ^2^ ***: *p* < 0.01, **: *p* < 0.05, *: *p* < 0.1.

## Data Availability

Data are available from a publicly accessible repository: (1) Demographic Statistics, Official website of Macao Bureau of Statistics, 2022; available from: https://www.dsec.gov.mo (18 December 2022). (2) Statistical Yearbook of Macau Health Bureau, available from: https://www.ssm.gov.mo (18 December 2022).
